# The Role of Early Rehabilitation in Treatment of Acute Pulmonary Embolism—A Narrative Review

**DOI:** 10.3390/jcm14176230

**Published:** 2025-09-03

**Authors:** Kamil Salwa, Karol Kaziród-Wolski, Dorota Rębak, Janusz Sielski

**Affiliations:** 1 Intensive Cardiac Care Unit, Świetokrzyskie Cardiology Center, 25-736 Kielce, Poland; kamil.salwa@wszzkielce.pl (K.S.); karol.kazirod-wolski@ujk.edu.pl (K.K.-W.); 2 Institute of Medical Sciences, Collegium Medicum, Jan Kochanowski University in Kielce, 25-317 Kielce, Poland; 3 Institute of Health Sciences, Collegium Medicum, Jan Kochanowski University in Kielce, 25-317 Kielce, Poland

**Keywords:** acute pulmonary embolism, respiratory rehabilitation, post-pulmonary embolism syndrome, exercise therapy, anticoagulation treatment, functional recovery

## Abstract

**Background/Objectives**: Pulmonary embolism (PE) is a life-threatening condition that frequently results in persistent exertional dyspnea, reduced exercise tolerance, and psychological distress, even after acute-phase management. Despite growing recognition of post-PE impairments, structured early rehabilitation remains underutilized. This narrative review aims to evaluate current evidence on the role, components, and outcomes of early rehabilitation strategies following acute PE. **Methods**: Following Preferred Reporting Items for Systematic Reviews and Meta-Analyses (PRISMA) methodology, a comprehensive literature search was conducted across PubMed, Embase, Scopus, Web of Science, Google Scholar, and the Cochrane Library. Articles published between 2020 and 2025 were screened for relevance to early rehabilitation in PE patients. Inclusion criteria prioritized randomized controlled trials, prospective studies, meta-analyses, and systematic reviews. Study quality was assessed using Cochrane, Newcastle–Ottawa, and AMSTAR 2 tools. **Results**: Out of 306 records, 158 studies were included. Early pulmonary rehabilitation—including aerobic and resistance exercise, inspiratory muscle training, and psychological support—demonstrated improvements in functional capacity, dyspnea, and quality of life, without adverse effects. Supplementation with omega-3 fatty acids and vitamin D may further mitigate thromboembolic risk via anti-inflammatory pathways. However, evidence remains predominantly observational, with limited high-quality data addressing timing, dosage, and patient stratification. **Conclusions**: Early, individualized rehabilitation appears safe and potentially beneficial in improving recovery and limiting complications after acute PE. Nonetheless, the absence of robust randomized trials underscores the urgent need for hypothesis-driven research to establish standardized, evidence-based rehabilitation protocols and guidelines tailored to risk stratification and patient phenotype, so as not to prolong recovery time and keep survivors from becoming disabled.

## 1. Introduction

Pulmonary embolism (PE) impairs pulmonary perfusion and respiratory function due to various intravascular obstructions. Common symptoms include chest pain, hemoptysis, and dyspnea. The World Health Organization (WHO) Mortality Database (2013–2015) shows that PE causes about 10/1000 deaths annually in females (1%) and 5/1000 in males (0.5%) aged 15–55 across Europe [1]. Advances in diagnostics have increased PE detection rates. Although clinical recovery typically occurs within 3–6 months, many survivors suffer from persistent symptoms, reduced quality of life, and impaired physical function. Historically, focus remained on diagnosis and acute treatment, overlooking post-acute rehabilitation and chronic sequelae [2]. In 2019, the European Society of Cardiology recommended a structured post-discharge strategy for symptomatic patients, including physical reconditioning, comorbidity management, lifestyle education, and risk factor modification [3]. Recently, pulmonary rehabilitation has gained recognition for non-obstructive lung diseases like PE [4,5]. PE patients show diverse respiratory and motor impairments [6]. A Chinese study demonstrated benefits of a six-week low-intensity exercise program in acute PE patients [7]. However, no standardized early rehabilitation protocols exist, and few studies assess long-term safety and efficacy. Exertional dyspnea and fatigue remain the most reported post-PE symptoms. Further complications may include post-thrombotic syndrome (with deep vein thrombosis), post-embolic pulmonary hypertension, or recurrent embolism [8].

Recent epidemiological studies highlight that idiopathic pulmonary embolism (IPE)—defined as acute PE without transient or persistent risk factors such as malignancy, surgery, immobility, thrombophilia, or systemic inflammatory disease—represents only 6–13% of all PE cases. Despite thorough diagnostic assessments, including thrombophilia testing and malignancy screening, many remain cryptogenic. IPE occurs more often in younger, otherwise healthy adults, with several cohorts noting a slight female predominance. Proposed mechanisms include occult prothrombotic states or subclinical endothelial dysfunction. Notably, recurrence risk is higher than in provoked events, justifying extended anticoagulation in selected patients. However, the low incidence and diagnostic complexity restrict large-scale, population-based analyses, and IPE remains underrepresented in registries.

In daily clinical practice, by contrast, acute PE more frequently arises as a complication of other conditions, particularly acute exacerbation of chronic obstructive pulmonary disease (AECOPD), severe pneumonia, or post-surgical states. Accordingly, this review addresses both isolated and secondary PE. The distinction is clinically significant: secondary PE requires rehabilitation strategies tailored to the underlying illness—integrating respiratory care, postoperative recovery, or critical care rehabilitation—whereas isolated IPE is managed primarily with anticoagulation [9,10,11,12,13,14,15].

## 2. Materials and Methods

**Figure 1:**
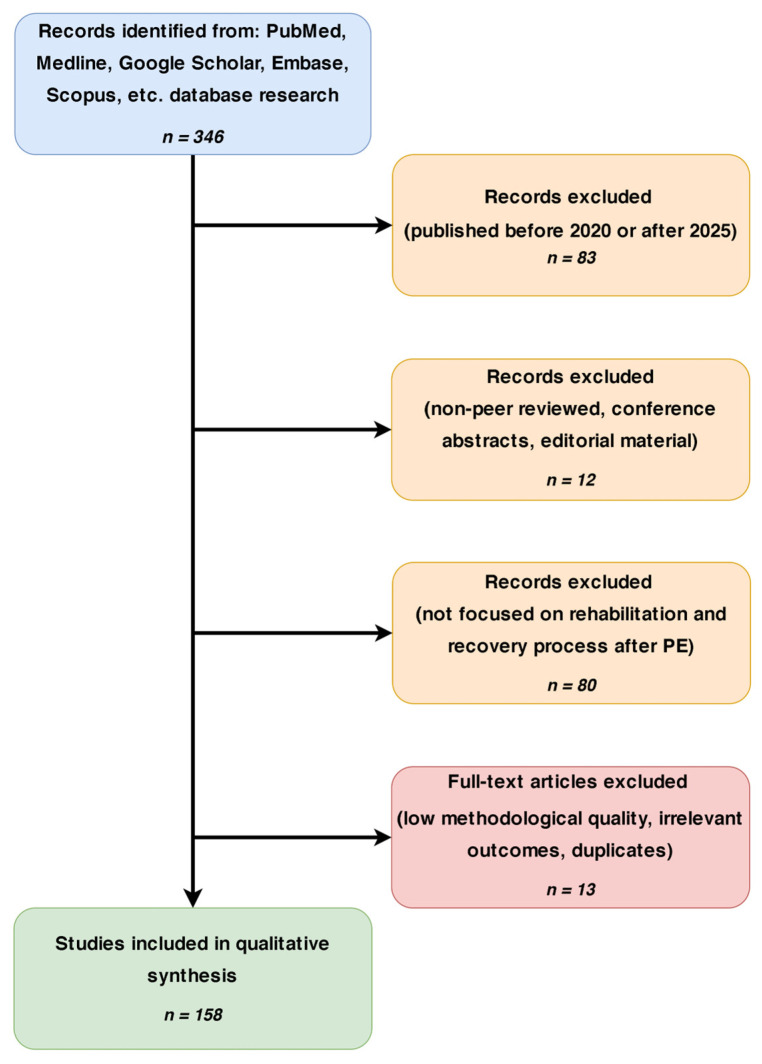
PRISMA-based flowchart illustrating the study selection process for the comprehensive review.

A review was conducted per PRISMA guidelines. A comprehensive search of PubMed/MEDLINE, Embase, Scopus, Web of Science, Google Scholar, and the Cochrane Library identified studies from 2020 to 2025 on early rehabilitation after acute PE. Search terms included “early rehabilitation”, “pulmonary embolism”, “physical therapy”, “exercise therapy”, “supplementation”, “anticoagulation treatment”, “early ambulation”, and “pulmonary rehabilitation”, combined with Boolean operators. Original studies were prioritized: randomized controlled trials (RCTs), observational and cohort studies, clinical trials, meta-analyses, and systematic reviews focused on early post-PE rehabilitation. Due to limited high-quality data, all eligible full texts were thoroughly analyzed. Study quality was evaluated using the Cochrane Risk of Bias Tool (RCTs), Newcastle-Ottawa Scale (observational/cohort), and AMSTAR 2 (systematic reviews/meta-analyses). Of 306 eligible records, 118 were included in the final synthesis. The procedure for identifying and selecting publications is illustrated in the algorithm shown in Figure 1. The specific inclusion and exclusion parameters applied in this review are presented in Table 1.

## 3. Results

### 3.1. Pathogenesis

Venous thromboembolism (VTE) comprises deep vein thrombosis and PE, arising from inherited or acquired disruptions of venous flow [16]. PE typically follows embolic occlusion of the pulmonary artery by thrombi from lower-limb deep veins, impairing pulmonary perfusion and right-heart function [17]. Inherited risks include factor V Leiden, activated protein C resistance, prothrombin G20210A, protein S insufficiency, and antithrombin deficiency. Acquired risks involve surgery, pregnancy, cancer, aging [2], and severe trauma, lower-limb fractures, arthroplasty, or spinal cord injury [3].

### 3.2. Health Implications of Pulmonary Embolism

The sequelae of PE significantly diminish health-related quality of life and impose considerable economic burden on healthcare systems. PE may induce persistent adverse outcomes that hinder reintegration into routine life activities for extended periods. Residual physical symptoms—such as exertional dyspnea, vertigo, thoracic pain, and exercise intolerance—can persist chronically, even in the absence of radiologically detectable thrombotic remnants [18]. Beyond somatic manifestations, a substantial proportion of patients exhibit psychological disturbances, including heightened anxiety and sleep dysregulation, collectively contributing to diminished motivation for re-engagement in routine physical and social activities [19].

#### 3.2.1. Physical Disfunctions

A prospective cohort analysis involving individuals diagnosed with PE demonstrated that nearly half exhibited exercise intolerance, defined by a predicted peak oxygen and elevated pulmonary arterial pressure. Predictably, diminished physical performance correlated with significantly lower health-related quality of life, increased dyspnea, and impaired ambulation capacity. Functional decline observed post-PE is predominantly attributed to physical deconditioning. Additionally, fear of recurrent thromboembolism frequently inhibits patients from resuming physical activity or engaging in any exercise programs [18].

PE leads to increased pulmonary vascular resistance, right ventricular dysfunction, V/Q mismatch, and inflammatory activation. Post-PE rehabilitation should assess how targeted physical and respiratory training may modulate these disturbances.

#### 3.2.2. Increased Pulmonary Vascular Resistance (PVR)

Pulmonary artery obstruction in PE redistributes blood flow, raising PVR and straining the right ventricle. Exercise may improve endothelial function and reduce PVR via shear stress, nitric oxide release, and vascular remodeling. However, evidence on its direct post-PE effects remains scarce, necessitating further clinical trials [20,21,22,23,24].

#### 3.2.3. Right Ventricular (RV) Dysfunction

Increased PVR places substantial strain on the right ventricle, leading to reduced output and signs of right heart failure. In chronic pulmonary hypertension, supervised exercise improves RV function via enhanced myocardial efficiency and vascular remodeling. While these benefits may apply to post-PE care, evidence in the acute phase is limited, highlighting the need for prospective studies on safety and efficacy of RV-targeted rehabilitation [25,26,27,28,29,30,31].

#### 3.2.4. Ventilation-Perfusion (V/Q) Mismatch

PE causes V/Q mismatch and hypoxemia due to impaired perfusion despite preserved ventilation. This gas exchange deficit is a critical focus of post-PE rehabilitation. Techniques like Active Cycle Breathing (ACBT) improve ventilation and airway clearance in other lung diseases, but their role in correcting V/Q mismatch after PE remains unclear. Rigorous studies are needed to define their efficacy and integration into early rehabilitation [32,33,34,35,36,37,38].

#### 3.2.5. Inflammation

Acute PE triggers systemic and local inflammation, worsening lung injury and impairing gas exchange. Elevated cytokines like Interleukin-6 (IL-6) and TNF-α contribute to both acute damage and delayed recovery. Moderate exercise reduces inflammation by modulating cytokine profiles and enhancing endothelial repair. However, its effect on post-PE inflammation remains unstudied, warranting further research on exercise-based rehabilitation and its clinical impact [39,40,41,42,43,44,45,46].

#### 3.2.6. Psychological Morbidity and Life Outcomes

A significant proportion of PE patients experience reduced quality of life, ongoing fatigue, and psychological distress [47,48]. Functional limitations are closely linked to decreased physical performance and contribute substantially to impaired quality of life [48]. While gradual improvement is common during the first year post-PE, a Canadian prospective study identified female gender, high body mass index, and early exercise intolerance as key predictors of poorer recovery trajectories [18]. Similarly, a German study confirmed quality of life improves within a year but is hindered by female sex, existing cardiopulmonary disease, and elevated body mass index [49]. Many PE survivors also seek psychological support during recovery [48]. A joint report by the European Society of Cardiology and the European Respiratory Society highlighted the importance of assessing long-term PE outcomes and recommended incorporating patient-reported outcome measures [50].

Psychological morbidity remains a major determinant of quality of life and functional recovery following acute pulmonary embolism, with patients frequently reporting persistent anxiety, depressive symptoms, and reduced confidence in physical exertion. Evidence suggests that structured behavioral change frameworks, long embedded in comprehensive cardiac rehabilitation programs, are equally applicable in the context of PE to address barriers to exercise adherence and promote sustainable lifestyle modification. Integrating such frameworks into PE-focused rehabilitation protocols ensures that psychological and behavioral outcomes are prioritized alongside cardiopulmonary recovery [51].

One of the most promising instruments in this context is the recently validated Coronary Heart Disease-Perceived Exercise Barriers Scale (CHD-PEBBS), which quantifies patients’ perceptions of physical, emotional, and logistical obstacles to physical activity. Although originally designed for coronary heart disease cohorts, its domains—fear of exacerbating symptoms, lack of confidence in exercise tolerance, and limited external support—map closely onto the concerns observed in PE survivors. The use of CHD-PEBBS in this population provides a structured tool to individualize psychological counseling, identify modifiable barriers, and integrate behavioral interventions with cardiopulmonary rehabilitation strategies [52].

Formal psychological support, including cognitive-behavioral therapy and psychoeducational counseling, has been shown to significantly improve adherence to exercise-based rehabilitation in patients with cardiovascular and pulmonary conditions. Recent meta-analyses demonstrate that embedding psychological support within rehabilitation programs not only reduces anxiety and depressive symptom burden but also increases long-term adherence to structured physical activity regimens, thereby enhancing functional outcomes and health-related quality of life [53].

The incorporation of validated instruments such as CHD-PEBBS within a multidisciplinary rehabilitation framework therefore strengthens the comprehensiveness of PE management, bridging current gaps between physical recovery and psychological resilience.

#### 3.2.7. Heterogeneity of the Pulmonary Embolism Population

PE is a clinically diverse condition affecting patients with varying demographic and clinical characteristics. Factors such as age, sex, malignancy, cardiovascular disease, obesity, and genetic predisposition influence its presentation and prognosis. Emerging data highlight distinct features in subgroups like pediatric patients and reveal gender-related and obesity-related differences, emphasizing the complexity of PE management and rehabilitation [54,55,56,57,58,59,60,61,62].

#### 3.2.8. Risk Stratification in Acute PE

Accurate initial risk stratification is pivotal in guiding both therapeutic and rehabilitative decisions. As outlined in the 2019 European Society of Cardiology (ESC) guidelines, patients with acute PE are classified into three principal risk categories:

- **High risk:** Defined by hemodynamic instability, including sustained hypotension (systolic blood pressure < 90 mmHg for >15 minutes) or cardiogenic shock.
- **Intermediate risk:** Hemodynamically stable patients exhibiting right ventricular dysfunction on imaging or elevated cardiac biomarkers (e.g., troponin).
- **Low risk:** Hemodynamically stable patients without RV dysfunction or biomarker elevation [63,64,65,66,67,68,69,70,71,72]

### 3.3. Pharmacological Treatment Strategies

Anticoagulation remains the primary treatment for acute PE, aimed at preventing thrombus extension and recurrence. The 2019 ESC guidelines recommend at least three months of therapy for all PE patients. Direct oral anticoagulants (DOAC), such as apixaban and rivaroxaban, are preferred due to their efficacy, safety, and predictable pharmacokinetics. In patients with contraindications to DOACs, vitamin K antagonists (VKA) are acceptable alternatives, typically preceded by low-molecular-weight heparin (LMWH) bridging until therapeutic INR is achieved. While European standards guide clinical practice, drug access and physician preference can influence treatment decisions. LMWH is often used in the early phase, especially in hospitalized patients. DOACs, however, remain the first-line option for most. Therapy duration should reflect individual risk profiles. For transient risk factors, a three-month course is usually sufficient. In unprovoked PE or persistent risk, extended therapy may be warranted, guided by bleeding risk. Reduced-dose DOACs beyond six months may offer continued protection with lower bleeding risk, though careful patient selection is essential. Effective PE management requires adherence to evidence-based protocols and individualized anticoagulant strategies based on thrombotic and hemorrhagic risk. In Poland, as globally, the overarching therapeutic objective remains the optimization of clinical outcomes through the rational application of modern anticoagulant agents, tailored to drug accessibility and the specific characteristics of the national patient population:

- **Low-molecular-weight heparins (LMWHs)** achieve therapeutic anticoagulant levels more rapidly than VKAs such as warfarin, a factor of critical importance in the early phase of treatment.
- **Time to therapeutic anticoagulation** is a key determinant of clinical outcomes, with expedited attainment of effective drug levels associated with a reduced risk of recurrent PE and VTE.
- **LMWHs** are preferred during the initial treatment phase, particularly in patients with active malignancy or those with elevated bleeding risk, due to their predictable pharmacodynamics, subcutaneous administration, and favorable safety profile.
- **Weight-adjusted dosing strategies** validated in clinical trials have been shown to enhance the therapeutic efficacy of LMWHs, underscoring the importance of individualized, weight-based regimens in optimizing anticoagulation outcomes [73,74,75,76,77,78,79,80,81,82,83,84,85].

### 3.4. Pulmonary Rehabilitation

**Figure 2:**
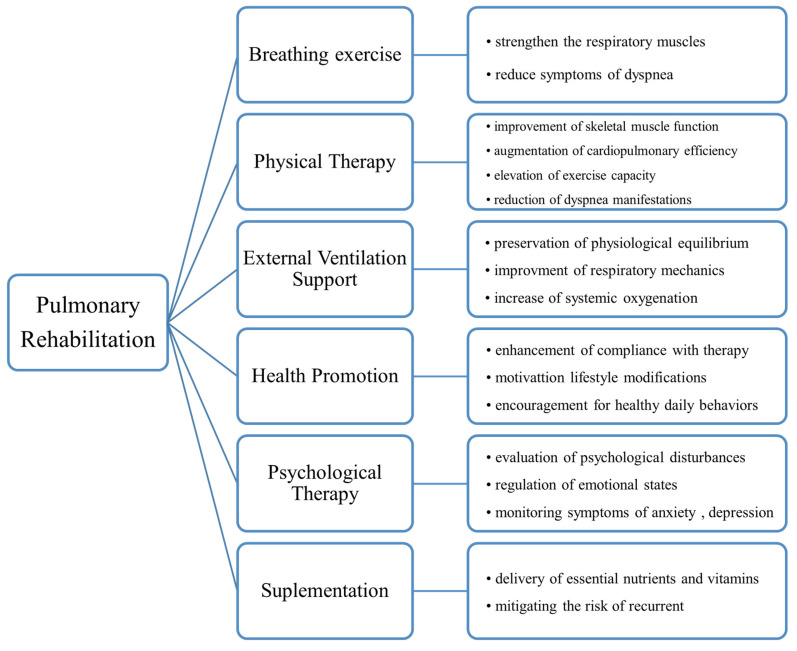
Pulmonary rehabilitation protocol for the care plan of pulmonary embolism.

Pulmonary rehabilitation constitutes a multidisciplinary intervention strategy encompassing a range of components, notably exercise conditioning, health prophylaxis, and behavioral modification, among other supportive measures [86]. The primary aims of pulmonary rehabilitation are to improve both physiological and psychological functioning in individuals with acute and chronic respiratory disorders and to facilitate sustained health optimization [87]. Figure 2 presents a schematic differentiation of the most important spheres of the early rehabilitation process in patients with PE.

#### 3.4.1. Physical Conditioning

Physical conditioning improves peripheral muscle function, cardiopulmonary response, and exercise tolerance, while reducing dyspnea. Modalities include interval and resistance training. Individualized programs are essential for feasibility, adherence, and long-term effectiveness. Training may target upper- and lower-limb strength. Pulmonary Rehabilitation Guidelines by the American Thoracic Society and European Respiratory Society emphasize lower-limb muscle training to reduce dyspnea and improve functional capacity [2]. Upper-limb training helps alleviate fatigue; thus, combined limb training is recommended for optimal results. Physiotherapists must tailor regimens to functional status and clinical needs, using both resistance and endurance components. Interval sessions may involve 4–8 bouts of 30 seconds to 4 minutes at moderate intensity, guided by perceived dyspnea (Borg scale 6–7). Progression involves increasing intervals and reducing rest. Resistance training should prioritize high-load, low-repetition strategies—about 5 RM per set—to minimize breathlessness. Lower-limb work targets large muscles, notably quadriceps, with 3–4 sets at 70–95% 1 RM. Upper-limb training uses 65–75% 1 RM, with 10–12 reps over 2–3 sets. Programs should also include exercises for posture and flexibility [88]. Early mobilization during hospitalization helps prevent muscle loss, deconditioning, and thromboembolic recurrence. Customized exercise improves circulation, reduces cardiac load, enhances respiration, and may lower ventilatory support needs. It also supports mental health, improves quality of life, boosts therapy engagement, and promotes metabolic recovery.

#### 3.4.2. Respiratory Exercises

Pulmonary exercises are central to pulmonary rehabilitation, focusing on strengthening ventilatory muscles. Inspiratory load-based training engages accessory muscles and the diaphragm, increasing endurance and contractile strength, thereby improving ventilatory efficiency. Evidence shows such training raises maximal inspiratory pressure and tidal volume in chronic pulmonary disease, enhancing muscle performance, exercise capacity, and quality of life [89,90]. For PE patients, physical therapists may use targeted inspiratory muscle training or resistance devices to boost endurance and reduce dyspnea [91]. Structured respiratory exercises—diaphragmatic breathing, nasal inspiration, and abdominal contraction—are essential for restoring pulmonary function. Instrumental techniques, especially in acute and post-acute phases, benefit patients with post-PE dysfunction by improving respiratory parameters. Programs using deep breathing, diaphragmatic activation, and pursed-lip exhalation aim to strengthen inspiratory muscles and enhance ventilation. Instrumental methods employ devices like incentive spirometers, positive expiratory pressure tools, and inspiratory trainers to optimize lung expansion and airway clearance. These approaches improve lung volumes, respiratory muscle function, and pulmonary hygiene, showing benefit in PE populations [92]. Respiratory–Swallow Training (RST), developed by Martin-Harris and McFarland, enhances coordination between respiration and swallowing. It trains patients to swallow during mid-to-low expiratory lung volume, using respiratory inductance plethysmography and nasal airflow monitoring via pressure transducers. Real-time visual feedback allows accurate timing of swallows. Data from thoracoabdominal movement and nasal pressure determine respiratory phase and lung volume, with swallow detection manually verified and validated offline. Current work seeks to automate analysis, improving detection accuracy and reliability of respiratory metrics at swallowing onset [93]. Respiratory training is vital in early PE rehabilitation. It improves lung mechanics, prevents atelectasis, reduces respiratory load, enhances gas exchange, and supports earlier mobility while decreasing ventilatory support needs, including NIV. These exercises ease dyspnea, encourage sustained behavioral change, reduce anxiety, and help psychological adjustment.

#### 3.4.3. External Ventilation Support

External ventilation support (EVS) is widely used in pulmonary rehabilitation to stabilize physiology, improve lung mechanics, and enhance systemic oxygen delivery [94]. However, positive pressure ventilation strains the right ventricle (RV) by lowering venous return and RV preload with each breath. Positive end-expiratory pressure further increases this effect. Pulmonary vascular resistance (PVR) rises with extreme lung volumes and altered blood flow distribution, elevating RV afterload. This can reduce right ventricular stroke volume (RVSV), left ventricular preload, and cardiac output, potentially causing hypotension and circulatory collapse [95]. To prevent these effects, mechanical ventilation should use tidal volumes of 6–8 mL/kg ideal body weight and cautious positive end-expiratory pressure, especially in RV dysfunction [95]. A new home-based EVS option offers greater accessibility and supports long-term rehabilitation needs [2]. Non-invasive ventilation (NIV) and continuous positive airway pressure CPAP are applied in acute PE patients with severe hypoxemia and ventilatory failure. Early implementation stabilizes respiratory status, enabling more effective breathing exercises and increasing comfort. Improved ventilation supports earlier physical activity, promotes lifestyle change, enhances nutrient absorption and cognition, and strengthens psychological recovery.

#### 3.4.4. Health Prophylaxis

Health prophylaxis constitutes a structured, methodically organized process aimed at informing patients about the pathophysiology of their condition, strategies for preventing recurrent embolic events, and the therapeutic importance of pulmonary rehabilitation within the broader continuum of care [86]. Prophylaxis facilitates patient understanding of therapeutic strategies and disease-specific precautions, while enhancing awareness of respiratory physiotherapy and structured exercise training. Furthermore, it promotes adherence to pharmacologic regimens and encourages the adoption of health-promoting behaviors, contributing to the attenuation of pulmonary function decline over time [96]. Accordingly, the provision of comprehensive health prophylaxis is essential to enhance therapeutic adherence, encourage modification of detrimental lifestyle factors, and promote the establishment of sustainable health-supportive behaviors in everyday living [97]. Health education targeting modifiable risk factors—such as obesity, smoking, and inactivity—is vital in early PE rehabilitation. Promoting healthy diets and regular exercise improves physical and respiratory function, accelerating recovery. Awareness of nutrition and supplementation supports physiological healing. Lifestyle education also reduces stress and enhances disease control, boosting psychological therapy and rehabilitation outcomes.

#### 3.4.5. Psychological Support

Prolonged illness often leads to psychological disturbances, with patients reporting depressive symptoms, irritability, and ongoing somatic complaints [98]. In acute PE, psychological distress arises from the life-threatening nature of the event. Even after stabilization, many survivors experience persistent fear of recurrence, contributing to long-term psychological imbalance and behavioral change. A long-term observational study in acute PE patients found frequent behavioral changes, reduced physical activity, and symptoms of post-traumatic stress. A nested qualitative analysis explored early recovery experiences, emotions, and thought patterns to inform strategies for restoring daily function and activity. In the hospital phase, symptoms should be gradually alleviated [2]. Pulmonary rehabilitation should include support for emotional regulation through structured training in emotional awareness and affective response modulation, aiming to improve resilience and outcomes [99]. Psychological care is critical after PE due to the high risk of anxiety, depression, and post-traumatic stress. Addressing these issues improves acceptance, supports engagement in physical and respiratory therapy, and accelerates recovery. Better mental health fosters sustainable lifestyle changes and adherence to supplementation. It may also reduce perceived dyspnea, lower ventilatory support needs, and improve overall prognosis.

#### 3.4.6. Supplementation

**Figure 3:**
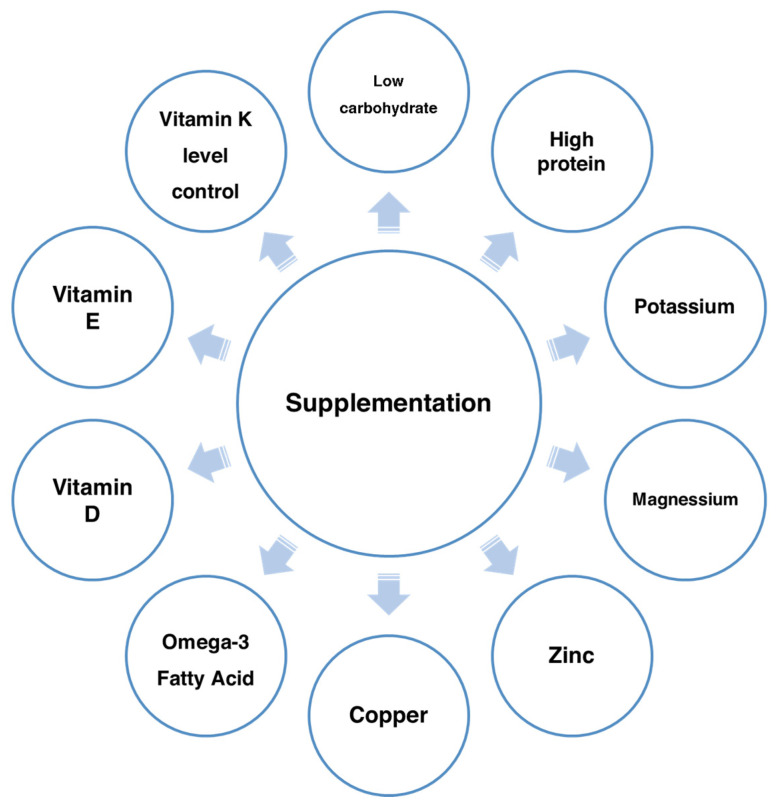
Components of dietary supplementation for a patient with acute PE.

Malnutrition is frequent in PE and worsens prognosis, making early nutritional intervention important. Diets with adequate protein and key micronutrients help preserve muscle and respiratory function, though PE-specific data are limited [2]. Omega-3 fatty acids may lower risk of PE, DVT, and mortality; daily intake and postoperative supplementation reduced events in some studies, while others reported no association, showing inconsistent results [100,101,102]. Zinc and copper support coagulation and vascular integrity, but imbalance may promote thrombosis [103]. Vitamin D influences immunity and coagulation, with deficiency linked to higher thrombogenicity and poorer PE outcomes, though evidence for prevention remains inconclusive [104,105,106]. Screening and supplementation may benefit high-risk patients [104]. Vitamin K deficiency can cause hemorrhage, and its metabolism may be impaired by antibiotics or vitamin E; monitoring is recommended in PE [107]. Omega-3s and vitamin D may reduce recurrence via anti-inflammatory and antithrombotic actions, but data are mostly observational. Adequate intake of vitamin D, magnesium, iron, and B vitamins supports recovery and adherence. Figure 3 summarizes key supplementation components for PE patients.

In summary, effective pulmonary rehabilitation for patients with PE necessitates interdisciplinary coordination to deliver comprehensive interventions encompassing disease management, symptom control, preservation of respiratory function, mental support, and nutritional optimization. According to the American College of Chest Physicians and the American Association of Cardiovascular and Pulmonary Rehabilitation, pulmonary rehabilitation is structured into three levels of therapeutic intensity: Level A includes core exercise training; Level B encompasses external ventilation support, respiratory muscle training, and structured health education; and Level C comprises nutritional supplementation. Clinicians are advised to implement tailored, patient-specific strategies [2].

Rehabilitation following acute pulmonary embolism lacks robust randomized evidence, so many strategies described here are extrapolated from related populations, particularly chronic obstructive pulmonary disease (COPD) and chronic thromboembolic pulmonary hypertension (CTEPH). The authors acknowledge these limitations. Nonetheless, COPD and CTEPH studies offer important insights into pulmonary rehabilitation, exercise training, and inspiratory muscle conditioning, as both share mechanisms of exercise limitation—ventilatory inefficiency, impaired gas exchange, and right ventricular strain—highly relevant after acute PE [108].

Nevertheless, caution must be exercised in transferring these strategies wholesale into acute PE care. COPD patients often undergo long-term, stable rehabilitation with predictable trajectories, whereas acute PE survivors may experience abrupt hemodynamic instability, anticoagulation-related bleeding risks, and evolving right heart recovery [3]. Therefore, while extrapolation is pragmatic in the absence of large-scale PE-specific rehabilitation trials, it is accompanied by the need for careful individualization, stepwise intensity adjustment, and rigorous safety monitoring.

Inspiratory muscle training, for instance, has demonstrated efficacy in improving functional capacity and reducing dyspnea in COPD and CTEPH patients, yet its role in acute PE remains underexplored [109].

In extrapolating such interventions, clinicians must consider both the temporal context (acute recovery vs. chronic adaptation) and the pathophysiological differences (embolism-induced pulmonary vascular obstruction vs. fixed airway or vascular remodeling). Similarly, structured exercise protocols validated in COPD cohorts, particularly low-to-moderate intensity aerobic regimens and interval training, provide a logical foundation for PE rehabilitation but require modification to account for anticoagulation, wound healing after surgery, or concurrent oncology treatment [110].

The absence of robust PE-specific data underscores the urgent need for targeted clinical trials. However, waiting for such evidence before implementing any form of rehabilitation risks delaying care in a high-morbidity population. Extrapolated approaches thus serve as interim, evidence-informed strategies that prioritize patient safety while promoting functional recovery. This pragmatic adaptation is consistent with international guideline methodology, which often relies on indirect evidence when high-quality disease-specific data are lacking [111].

By explicitly labeling these recommendations as extrapolations and clarifying their conditional applicability, we address the reviewer’s concern and highlight the research gaps in this field. Importantly, this transparency ensures that clinicians interpret the recommendations within their appropriate context and apply them with individualized caution. Future studies specifically investigating structured rehabilitation in acute PE populations are warranted to validate or refine these provisional strategies [18].

Table 2 presents a visual summary of the extrapolation of protocols and improvement techniques from publications on other disease syndromes with similar complications.

#### 3.4.7. Telehealth-Enabled Rehabilitation Approaches

The Coronavirus Disease 2019 (COVID-19) pandemic hastened the integration of telehealth-based rehabilitation, which has proven effective in enhancing both accessibility and adherence to cardiac and pulmonary programs. For patients with cardiopulmonary disease, including post-PE recovery, telehealth enables structured exercise supervision, remote physiological monitoring, and educational support without in-person attendance. This digital model reduces barriers of distance, mobility, and infection risk, while maintaining continuity of evidence-based rehabilitation [122].

Recent randomized trials and meta-analyses demonstrate that telehealth rehabilitation yields outcomes comparable to, and sometimes surpassing, traditional programs in exercise capacity, health-related quality of life, and psychological well-being. Integration of mobile apps, wearable monitors, and video-based sessions allows personalized exercise prescription with real-time feedback. These models are especially valuable for post-PE patients, where continuous oversight of anticoagulation, exercise safety, and adherence is essential [123].

Hybrid models that combine in-person initiation with remote follow-up have become increasingly adopted, offering the advantages of baseline face-to-face assessment while maintaining engagement through telemonitoring. These approaches lower attrition, improve satisfaction, and expand access to multidisciplinary teams including physiotherapists, psychologists, and specialist nurses. Post-COVID, telehealth frameworks are now embedded in cardiac rehabilitation guidelines, and extending them to PE survivors is a logical and feasible means of broadening participation in structured recovery [124].

#### 3.4.8. Baseline Factors Influencing Early Rehabilitation

In the early hospitalization phase, rehabilitation planning must be individualized based on specific patient characteristics:

- **Hemodynamic status:** High-risk patients require full hemodynamic stabilization prior to initiating any rehabilitation efforts.
- **Respiratory function:** The presence and severity of hypoxemia or dyspnea dictate the feasibility and type of respiratory interventions.
- **Comorbidities:** Conditions such as chronic obstructive pulmonary disease (COPD) or heart failure may limit exercise capacity and influence rehabilitation intensity.
- **Age and baseline functional status:** Older adults and individuals with preexisting mobility limitations necessitate adapted rehabilitation protocols.

#### 3.4.9. Rehabilitation Strategies According to Risk Level

- **High risk:** Focus remains on clinical stabilization; rehabilitation is deferred until hemodynamic parameters normalize.
- **Intermediate risk:** Upon stabilization, early initiation of supervised, moderate-intensity physical and respiratory therapy is appropriate, with continuous monitoring of vital signs.
- **Low risk:** Comprehensive rehabilitation may begin promptly and include aerobic and respiratory exercises, education on disease management, and strategies to address modifiable risk factors.

Table 3 presents abbreviated protocols for early rehabilitation of patients with PE depending on the risk stratification of disease recurrence.

#### 3.4.10. Early Rehabilitation of Patients with PE in ICU Settings

In ICU patients with pulmonary embolism, mobilization and rehabilitation must follow careful assessment of hemodynamic stability. It is contraindicated in persistent hypotension, uncontrolled tachyarrhythmias, or escalating vasopressor needs, as these markedly raise cardiopulmonary risk. Consensus guidelines recommend initiating activity only when mean arterial pressure is ≥65 mmHg without vasopressor escalation, oxygen saturation is >90% on stable ventilator settings, and no acute right ventricular decompensation is present. These criteria ensure mobilization promotes recovery rather than triggering deterioration [127].

Stepwise rehabilitation is crucial for mechanically ventilated patients to prevent immobility-related complications while considering ventilatory load. Protocols recommend a graded sequence: passive range-of-motion and in-bed cycling, followed by active-assisted exercises, bedside sitting, and eventually standing or ambulation as tolerated. Integrating physical therapy with respiratory interventions—such as inspiratory muscle training and graded spontaneous breathing trials—shortens ventilation duration, enhances independence, and reduces ICU-acquired weakness. A multidisciplinary team of physiotherapists, intensivists, and respiratory therapists is essential to adapt exercise intensity to respiratory mechanics and gas exchange capacity [125].

Monitoring is pivotal in protecting high-risk PE patients during early rehabilitation. Standardized tools—including the Borg scale for exertion, continuous SpO_2_, heart rate, and respiratory rate monitoring—offer real-time feedback on tolerance to mobilization. Interpreted alongside anticoagulation status and bleeding risk, these measures enable clinicians to adjust rehabilitation intensity and detect early decompensation. Evidence indicates that structured monitoring during mobilization reduces adverse events and supports safe functional recovery, especially in cardiopulmonary-fragile patients after acute PE [126].

#### 3.4.11. Differentiate Rehabilitation Approaches by Etiology and Comorbidity

In oncology patients with secondary pulmonary embolism, rehabilitation is challenged by bleeding risk, thrombocytopenia, and cancer-related cachexia. Exercise programs must balance anticoagulation safety with mobilization, typically using low-intensity aerobic activity, cautious resistance training, and strict platelet monitoring. Emerging evidence shows that integrated cardio-oncology rehabilitation lowers thromboembolic recurrence, reduces therapy-induced cardiopulmonary toxicity, and enhances quality of life, though training loads require individual adjustment in cytopenic states [128].

In post-surgical or trauma-related pulmonary embolism, rehabilitation timing is dictated by wound healing and tissue repair. Evidence indicates that mobilization begun once hemostasis and wound stability are secured reduces thromboembolic recurrence and improves function. Intensity must be cautiously titrated to avoid stressing surgical sites or fractures, while incorporating progressive ambulation and breathing therapy. Enhanced recovery protocols endorse a multidisciplinary strategy that balances the benefits of early mobilization with risks of bleeding or impaired healing [129].

In patients with advanced chronic lung disease or concomitant heart failure, rehabilitation is limited by ventilatory constraints and reduced cardiac output. Strategies emphasize inspiratory muscle training, cautious submaximal aerobic exercise, and interval regimens under strict cardiopulmonary monitoring. Evidence shows that adjusting intensity to oxygen saturation and hemodynamic responses prevents decompensation while improving functional capacity and dyspnea. Such individualized programs are essential to balance safety with therapeutic benefit [130].

In ICU patients with pulmonary embolism requiring mechanical ventilation or extracorporeal support, rehabilitation should follow a staged mobilization paradigm to mitigate ICU-acquired weakness and post-intensive care syndrome. Evidence supports early in-bed exercise, neuromuscular stimulation, and gradual progression to sitting and standing once stable, which reduce muscle wasting, aid ventilator weaning, and limit long-term disability. Multidisciplinary ICU rehabilitation has proven effective in restoring autonomy and preventing the sequelae of prolonged immobilization and critical illness [131].

#### 3.4.12. Individualization of Rehabilitation Parameters

Effective rehabilitation following PE requires a personalized approach grounded in the following:

- **Assessment of exercise tolerance:** Repeated functional testing allows for the adjustment of exercise intensity.
- **Monitoring of clinical symptoms:** Real-time observation of dyspnea, chest discomfort, and fatigue enables dynamic modifications to the rehabilitation plan.
- **Patient-centered care:** Incorporating patient preferences, motivation, and engagement significantly enhances adherence and therapeutic outcomes.

Rehabilitation after acute pulmonary embolism is a pivotal phase of care, targeting both cardiopulmonary recovery and long-term reduction in morbidity and recurrence. In idiopathic or primary PE, usually without major comorbidities, structured programs with graded aerobic training, diaphragmatic strengthening, and breathing control improve functional capacity, reduce post-thrombotic dyspnea, and ease psychological distress, including anxiety and fear of recurrence. Recent trials confirm that individualized pulmonary rehabilitation accelerates recovery and enhances diaphragmatic function in post-embolic patients [132].

In malignancy-associated PE, rehabilitation must align with oncological therapy. The emerging cardio–oncology rehabilitation model, endorsed by international consensus, emphasizes low- to moderate-intensity aerobic training, progressive resistance exercise, and tailored pulmonary physiotherapy to preserve independence amid chemotherapy-induced sarcopenia and cachexia. These strategies improve treatment tolerance, lower venous thromboembolic recurrence, and mitigate the cardiopulmonary toxicities of cancer regimens [128].

Following major surgery, particularly orthopedic and thoracic procedures, early mobilization protocols are essential for preventing recurrent venous thromboembolism and promoting musculoskeletal recovery. Physiotherapy-guided mobilization, incentive spirometry, and progressive ambulation have been linked to significant reductions in postoperative pulmonary complications, including secondary PE, while improving surgical outcomes. Evidence emphasizes the need to balance adequate mobilization to prevent thromboembolism with protection of surgical sites during early healing [133].

Patients with pulmonary embolism occurring in the context of trauma or intensive care present particular challenges due to critical illness, prolonged immobility, and systemic inflammation. Evidence increasingly supports early mobilization in the intensive care unit, including passive and active range-of-motion exercises, bedside cycling, and gradual progression from sitting to standing. These interventions have been shown to decrease thromboembolic complications, shorten the duration of intensive care, and improve functional outcomes at discharge, while maintaining hemodynamic stability [134].

In patients with chronic obstructive pulmonary disease or fibrotic lung disease who develop pulmonary embolism, rehabilitation must emphasize airway clearance, inspiratory muscle training, and exercise programs tailored to limited ventilatory reserve. Incorporation of pulmonary rehabilitation in this group has been shown to reduce dyspnea, improve oxygenation, and decrease readmission rates while addressing the combined impact of obstructive or restrictive pathology together with thromboembolic disease [135].

In patients with cardiovascular comorbidities, especially heart failure, rehabilitation should follow principles of cardiac rehabilitation, with cautious adjustment of aerobic workload, inclusion of resistance training, and close monitoring of hemodynamic responses. Incorporating cardiopulmonary rehabilitation in this group reduces exertional intolerance, improves ventricular function, and lowers the risk of recurrent embolic events by targeting systemic endothelial dysfunction and impaired venous return [130].

Enhanced recovery strategies in trauma and perioperative care show that a multidisciplinary approach combining early mobilization, pulmonary rehabilitation, nutritional optimization, and psychological support accelerates recovery and lowers the incidence of secondary pulmonary embolism. This evidence supports incorporating Enhanced Recovery After Surgery principles into the rehabilitation of pulmonary embolism patients across different clinical contexts, underscoring the importance of coordinated, team-based care [136].

In patients who develop pulmonary embolism during or following acute exacerbations of chronic obstructive pulmonary disease (AECOPD), rehabilitation strategies must account for the dual burden of persistent airflow limitation and the prothrombotic milieu associated with acute inflammatory exacerbations. Early initiation of structured pulmonary rehabilitation following stabilization from the exacerbation, with emphasis on inspiratory muscle training, airway clearance techniques, and progressive aerobic conditioning, has been shown to significantly reduce hospital re-admissions and improve exercise tolerance. Moreover, rehabilitation in this setting must be carefully staged to avoid precipitating further exacerbations, with particular attention to oxygen titration and close monitoring for hypercapnia in patients with chronic CO_2_ retention [137].

In patients with pulmonary embolism complicated by acute severe pneumonia, rehabilitation is particularly challenging as pneumonia decreases pulmonary reserve and intensifies systemic inflammation, fostering a hypercoagulable state. Management requires gradual progression of physical activity, pulmonary hygiene techniques, and respiratory physiotherapy to restore mucociliary clearance and prevent atelectasis. Evidence highlights that rehabilitation after severe pneumonia must be multidisciplinary, addressing not only respiratory recovery but also sarcopenia and deconditioning, which heighten the risk of recurrent thromboembolic events [138].

#### 3.4.13. The Role of a Multidisciplinary Team

Comprehensive rehabilitation after pulmonary embolism must extend beyond physical training and involve a multidisciplinary team. Physiotherapists play a central role by prescribing structured exercise, guiding stepwise mobilization, and delivering respiratory training adapted to the cardiopulmonary limitations of acute PE recovery. These interventions reduce deconditioning, enhance ventilatory efficiency, and lower the risk of recurrent thromboembolic events, reflecting benefits long established in cardiac and pulmonary rehabilitation programs [139].

Psychologists are equally important in managing the psychological morbidity common in pulmonary embolism survivors, including anxiety, depression, and post-thrombotic stress. Evidence shows that interventions such as cognitive-behavioral therapy and motivational interviewing improve adherence to rehabilitation and enhance long-term quality of life [140].

The use of validated behavioral instruments, such as perceived barriers scales, enables personalized support and promotes sustained engagement in rehabilitation pathways.

Nutritional optimization led by dietitians supports rehabilitation by addressing weight management, metabolic control, and micronutrient adequacy, all of which significantly influence cardiopulmonary outcomes. In cardiovascular populations, such interventions improve exercise tolerance and reduce inflammation, indicating similar benefits for patients recovering from pulmonary embolism [141].

Social workers contribute by addressing socioeconomic determinants of health, helping patients overcome barriers such as limited access to rehabilitation services, financial difficulties, and disrupted care continuity. Their involvement ensures better integration within healthcare systems, improving equity of access and participation in structured rehabilitation.

Experiences from other areas of cardiology confirm the importance of multidisciplinary rehabilitation models. Team-based programs in heart failure, ischemic heart disease, and post-surgical cardiac recovery have demonstrated improved functional outcomes, greater adherence, and higher patient satisfaction [142].

Extending these models to pulmonary embolism management is a logical and necessary step, uniting physical, psychological, nutritional, and social aspects of recovery within a cohesive rehabilitation framework.

#### 3.4.14. Critical Analysis of Exercise and Respiratory Training Recommendations

Current guidelines advise a cautious, individualized approach to exercise and pulmonary rehabilitation in PE recovery, based on comprehensive risk assessment and evaluation of hemodynamic and functional status. Although structured rehabilitation is effective in COPD and chronic thromboembolic pulmonary hypertension, direct evidence for its benefit in PE remains scarce. Extrapolated data indicate possible advantages, but the lack of PE-specific high-quality trials precludes definitive conclusions. Robust clinical studies are therefore required to establish the safety, efficacy, and optimal timing of exercise interventions, and to develop targeted, evidence-based rehabilitation protocols for this population.

### 3.5. Clinical Relevance of Pulmonary Physiotherapy

Appropriately structured respiratory rehabilitation has the potential to alleviate acute symptomatology associated with PE. However, the current body of research addressing pulmonary physiotherapy in this patient population remains limited in scope, with insufficient high-quality evidence guiding standardized protocols; long-term outcomes are shown structurally in Table 4.

A randomized clinical trial in acute VTE patients showed that, after 3 months, neither the exercise group nor the control experienced serious adverse events, confirming the safety of structured physical activity. The exercise group demonstrated significant improvements in physical activity and VO_2_max, indicating enhanced cardiopulmonary fitness [2]. Nopp et al. [7] reported that a 6-week multidisciplinary outpatient rehabilitation program significantly improved exercise capacity in 22 PE patients with persistent symptoms. In the Netherlands, a 12-week outpatient program combining sports and psychological counseling also effectively reduced symptoms in an observational cohort [4]. German researchers developed an individualized rehabilitation protocol for intermediate- and high-risk PE patients, based on clinical severity and baseline function. No serious adverse events occurred; in-hospital mortality was 0%, and 12-month mortality was 5.7%, supporting both safety and efficacy [2]. Rolving et al. [144] conducted a randomized trial assessing an 8-week home-based exercise program with nurse consultations in newly diagnosed PE patients. Although it showed no significant gains in exercise capacity or dyspnea, the intervention was safe and free of adverse events [2]. These results may reflect a lack of specialist supervision, insufficient motivation, low adherence, or minimal functional impairment during early hospitalization.

## 4. Insights and Perspectives

Pulmonary embolism (PE) is a common cardiovascular emergency and, as of 2015, ranked third in cardiovascular-related mortality in Europe [2]. Since then, detailed country-specific data on PE-related deaths remain limited. With advances in clinical care, the focus has shifted from acute management to long-term outcomes and post-acute strategies [145]. Although many patients return to daily activities within 6–12 months post-discharge, functional limitations and difficulty maintaining physical activity persist in a significant subset [2]. Clinical guidelines now emphasize the role of structured rehabilitation to address ongoing impairments and improve recovery [3]. Pulmonary rehabilitation has shown to reduce dyspnea, improve health status, and enhance exercise capacity in patients with PE [144], though standardized protocols and evaluation tools are still lacking. Current evidence supports its safety and effectiveness, with no reported adverse outcomes [143], and consistent improvements in functional performance and quality of life have been documented [146]. When pulmonary gains are limited, adjunctive approaches such as patient education and psychological support should be integrated to enhance overall outcomes [86]. In 2024, Azzarito et al. studied 225 patients approximately eight days post-PE, who completed a four-week inpatient cardiopulmonary rehabilitation program. Participants showed significant improvements in dyspnea and physical function, independent of acute-phase treatment and without adverse events [8]. Despite growing data, key questions remain unresolved: the optimal timing of rehabilitation initiation, appropriate training intensity relative to daily activity, duration needed to achieve benefit, and the potential value of additional therapeutic components. Broader research is required to define these parameters. Early rehabilitation during acute PE differs from that in chronic conditions due to rapidly changing hemodynamic, respiratory, and metabolic states, necessitating individualized, closely monitored protocols. Without timely mobilization, patients are at risk of worsening dyspnea, deconditioning, atelectasis, and anxiety. Early intervention aims to counteract these effects through coordinated physical, respiratory, nutritional, and psychological support. Initiating rehabilitation within seven days of hospitalization appears clinically justified in hemodynamically stable patients receiving therapeutic low-molecular-weight heparin (LMWH), usually effective within 48–72 hours. LMWH’s predictable pharmacokinetics and subcutaneous administration make it suitable for early mobilization. Once blood pressure and heart rate stabilize, patients may safely begin tailored exercises and respiratory training. Although direct evidence in PE is limited, data from related populations support early mobilization in reducing complications and enhancing outcomes. Therefore, early, supervised rehabilitation should be considered a key element of care for selected patients with acute PE [147,148,149,150,151,152,153,154,155,156,157,158].

### Limitations of Study

This narrative review, although methodically structured, is constrained by several critical limitations. The predominance of observational data and the paucity of randomized controlled trials restrict the capacity for causal inference and elevate the risk of bias. Considerable heterogeneity in study designs, rehabilitation protocols, and outcome measures undermines the comparability of findings and weakens the formulation of standardized recommendations. The concept of “early rehabilitation” lacks uniform operationalization across studies, complicating the delineation of optimal intervention timing and dosage. Moreover, the limited duration of follow-up in most studies precludes evaluation of long-term efficacy and sustainability. The geographic and institutional concentration of included studies limits external validity, particularly for high-risk or multimorbid populations who are underrepresented in the available literature. Publication bias remains a concern, as studies demonstrating positive outcomes are more likely to be published, further skewing the evidence base. Finally, the inherent subjectivity of narrative synthesis, in the absence of quantitative meta-analytic pooling, limits reproducibility and may influence interpretation. To advance clinical practice, future research must prioritize high-quality, multicenter randomized trials employing standardized rehabilitation protocols and stratified analyses to establish robust, evidence-based post-PE rehabilitation frameworks.

## 5. Conclusions

Pulmonary rehabilitation is a developing intervention for patients with PE, though current evidence is limited by a lack of randomized controlled trials. There is an urgent need for large-scale, prospective studies. Observational findings suggest rehabilitation programs, such as those implemented by the German Pension Fund, are safe and well-tolerated in post-PE populations. Patients recovering from PE are clinically heterogeneous and carry varying risks of recurrence. Preliminary evidence indicates that pulmonary rehabilitation may help mitigate Post-Pulmonary Embolism Syndrome (PPES). Despite persistent knowledge gaps, improvements in identifying suitable candidates, determining optimal timing, selecting appropriate modalities, and defining treatment duration are essential for enhancing long-term care. Additional studies are needed to clarify these areas. In conclusion, current data on early rehabilitation post-acute PE are scarce, inconsistent, and lack methodological rigor. Most available studies focus on delayed intervention, with a notable absence of high-quality trials in the early phase. Considering its potential to prevent long-term complications, early rehabilitation warrants hypothesis-driven research to examine its physiological effects. Key outcomes should include pulmonary vascular resistance, right ventricular function, ventilation–perfusion mismatch, and inflammation, using advanced diagnostics. Future trials must integrate personalized approaches based on risk assessment to establish evidence-based early rehabilitation protocols and improve patient outcomes.

## Figures and Tables

**Table 1:** Eligibility criteria for article selection in this narrative review.

Inclusion Parameters	Exclusion Parameters
Articles published (2020–2025)	Articles published before 2020 or after 2025
Editorial and peer-reviewed publications	Non-peer-reviewed works, editorials, letters, and abstracts without full text
Studies on rehabilitation and early rehabilitation after PE	Studies not directly or indirectly related to PE
Articles consistent with current evidence-based guidelines	Articles lacking adherence to current management or rehabilitation guidelines after PE
Original research: RCTs, cohorts, observational studies, structured reviews	Low-quality studies lacking defined design or analytical rigor
Articles published in English	Non-English version of publications
Full-text articles available for appraisal and synthesis	Articles without accessible full text or evaluable methodology

**Table 2:** Summary of rehabilitation interventions extrapolated from COPD, ILD, and CTEPH populations with their applicability and limitations in APE.

Intervention	Source Disease Population	Evidence Level *	Applicability to Acute PE
Aerobic exercise training [112,113]	COPD, CTEPH	Moderate: multiple RCTs, meta-analyses (2020–2024)	Conditional: may improve exercise tolerance, requires cautious intensity titration under anticoagulation
Inspiratory muscle training [110,114]	COPD, CTEPH	Moderate: RCTs, systematic reviews	Possible benefit for dyspnea and ventilatory efficiency; limited direct PE evidence
Interval training [115]	COPD	Moderate: RCTs, observational	Useful for graded recovery; safety in acute PE requires validation
Early mobilization (bed-to-chair, ambulation) [116,117,118]	ICU patients with critical illness (mixed)	Strong: meta-analyses, ICU rehabilitation studies	Applicable with hemodynamic stability; direct PE evidence lacking
Multidisciplinary pulmonary rehab programs [119,120,121]	COPD, interstitial lung disease, CTEPH	Strong for COPD/ILD; emerging for CTEPH	Framework applicable to PE, but must be adapted for acute and anticoagulated patients

* Evidence level as judged by number and quality of randomized controlled trials and systematic reviews available in the 2020–2025 literature.

**Table 3:** Risk-stratified early rehabilitation protocol for patients with APE: clinical pathway integrating initiation timing, therapeutic modalities, and monitoring criteria.

Risk Category of PE	Initiation of Rehabilitation	Therapeutic Modalities	Progression Criteria
**High risk** (massive, post-thrombolysis, ECMO, hemodynamic instability)	≥7–10 days post-diagnosis; only after full hemodynamic stabilization (MAP ≥ 65 mmHg, HR < 110 bpm, SpO_2_ ≥ 90% on FiO_2_ ≤ 0.6, stable anticoagulation, no active bleeding)	Respiratory muscles manual stimulation; Development of spatial body schema; Passive joint mobilization; Low-intensity inspiratory muscle training; Neurophysiological techniques; Psychological support; Delirium prevention.	Continuous ECG, invasive/non-invasive BP, SpO_2_, HR, Borg dyspnea scale; daily bleeding risk assessment. Progression allowed once hemodynamic and oxygenation remain stable ≥48 h; gradual transition to standing/active-assisted mobility if SpO_2_ ≥ 92% and no arrhythmias.
**Intermediate risk** (submassive, RV dysfunction but stable)	3–5 days post-diagnosis; after therapeutic anticoagulation stabilization and improved RV function on ECHO imaging	Active-assisted mobilization; Bedside sitting to standing; Corridor ambulation under active supervision; Breathing retraining (pursed-lip, diaphragmatic); Moderate inspiratory muscle training; Psycho-educational counseling.	SpO_2_ and HR telemetry, RR, Borg scale for dyspnea/exertion, INR or anti-Xa monitoring. Progression to ≥5–10 min corridor ambulation if Borg <5 and no desaturation >4% from baseline; Add resistance training with elastic bands if stable.
**Low risk** (no RV dysfunction, hemodynamically stable)	24–48 h post-diagnosis; after first therapeutic anticoagulation dose and no bleeding	Early ambulation; Progressive walking; Low-resistance training; Inspiratory muscle training; Airway clearance techniques; Psycho-educational support; Adaptive strategies.	HR and SpO_2_ pre/post activity, Borg scale, perceived exertion; anticoagulation monitoring. Escalate every 48–72 h by increasing intensity/duration; transition to formal outpatient pulmonary rehabilitation at discharge.

Abbreviations: ECMO—extracorporeal membrane oxygenation, ECHO—echocardiography, HR—heart rate, MAP—mean arterial pressure. Developed by synthesizing findings across prior studies [3,110,125,126].

**Table 4:** Current research on the health implications of pulmonary physiotherapy in patients with acute pulmonary embolism.

Authors	Year	Country	Type of Research	Specificity	Results
Cires-Drouet et al. [143]	2020	Netherlands	Prospective study	A structured 3-month physical conditioning regimen.	Exercise therapy at differentiated levels demonstrated safety following acute PE episodes.
Rolving et al. [144]	2020	Denmark	Randomized clinical trail	A short nursing-guided consultation combined with an 8-week home-based physical conditioning regimen.	No improvement was observed in physical performance or dyspnea symptoms. Furthermore, no additional adverse effects were recorded.
Nopp et al. [7]	2020	Austria	Prospective study	A structured rehabilitation program involving different types training lasting a minimum of 6 weeks.	The 6-minute walk test indicated enhanced outcomes. Significant positive motor function increases were also recorded. Additionally, 78% of patients exhibited improved health status during extended follow-up.
Boon et al. [4]	2021	Netherlands	Observational cohort study	A 12-week outpatient pulmonary rehabilitation program with consultations from a pulmonologist and physiotherapist.	Enhanced training intensity resulted in improvements in PE-specific quality of life, reduced fatigue, and better functional status.
Gleditsch et al. [88]	2022	Norway	Cohort sub-study	An outpatient pulmonary rehabilitation program supervised during 1-hour training sessions, twice a week, for 8 weeks.	CMR parameters were compared before and after the intervention. Both absolute RV global longitudinal strain and RV lateral longitudinal strain showed significant reductions.
Azzarito et al. [8]	2024	Italy	Prospective study	A 4-week inpatient cardiopulmonary rehabilitation program began 8 days following the pulmonary event.	All patients demonstrated improvements in both dyspnea and physical performance. No adverse effects related to the rehabilitation program were reported.

## Abbreviations

The following abbreviations are used in this manuscript:

ACBT	Active Cycle of Breathing Techniques
AECOPD	Acute Exacerbation of Chronic Obstructive Pulmonary Disease
CHD-PEBBS	Coronary Heart Disease—Perceived Exercise Barriers Scale
CO_2_	Carbon Dioxide
COPD	Chronic Obstructive Pulmonary Disease
COVID-19	Coronavirus Disease 2019
CPAP	Continuous Positive Airway Pressure
CT	Computed Tomography
CTEPH	Chronic Thromboembolic Pulmonary Hypertension
DOAC	Direct Oral Anticoagulant
ECMO	Extracorporeal Membrane Oxygenation
ECHO	Echocardiography
ESC	European Society of Cardiology
EVS	External Ventilation Support
FiO_2_	Fraction of Inspired Oxygen
HR	Heart Rate
ICU	Intensive Care Unit
IL-6	Interleukin-6
ILD	Interstitial Lung Disease
INR	International Normalized Ratio
IPE	Idiopathic Pulmonary Embolism
LMWH	Low-Molecular-Weight Heparin
MAP	Mean Arterial Pressure
NT-proBNP	N-Terminal Pro-B-type Natriuretic Peptide
NIV	Non-Invasive Ventilation
PE	Pulmonary Embolism
PPES	Post-Pulmonary Embolism Syndrome
PRISMA	Preferred Reporting Items for Systematic Reviews and Meta-Analyses
PUFA	Polyunsaturated Fatty Acids
PVR	Pulmonary Vascular Resistance
RM	Repetition Maximum
RST	Respiratory–Swallow Training
RV	Right Ventricle
RVSV	Right Ventricular Stroke Volume
SPECT	Single Photon Emission Computed Tomography
SpO_2_	Peripheral Capillary Oxygen Saturation
TNF-α	Tumor Necrosis Factor Alpha
UCI	Unidad de Cuidados Intensivos (Intensive Care Unit, Spanish)
V/Q	Ventilation/Perfusion
VKA	Vitamin K Antagonist
VTE	Venous Thromboembolism
WHO	World Health Organization
